# Pilot Study of Personalized Transcranial Magnetic Stimulation with Spectral Electroencephalogram Analyses for Assessing and Treating Persons with Autism

**DOI:** 10.3390/jpm14080857

**Published:** 2024-08-12

**Authors:** Milan T. Makale, Chad Nybo, Kenneth Blum, Catherine A. Dennen, Igor Elman, Kevin T. Murphy

**Affiliations:** 1 Department of Radiation Medicine and Applied Sciences, University of California San Diego, La Jolla, CA 92093, USA; 2 CrossTx Inc., Bozeman, MT 59715, USA; 3 Division of Addiction Research & Education, Center for Exercise Sports, Mental Health, Western University Health Sciences, Pomona, CA 91766, USA; 4 Department of Family Medicine, Jefferson Health Northeast, Philadelphia, PA 19114, USA; 5 Department of Psychiatry, Harvard University School of Medicine, Cambridge, MA 02215, USA; 6 Division of Personalized Neuromodulations, PeakLogic, LLC, Del Mar, CA 92130, USA

**Keywords:** repetitive transcranial magnetic stimulation, spectral EEG, autism spectrum condition (ASC), psychometric ASC tests

## Abstract

Autism spectrum condition (ASC) is a neurodevelopmental condition that is only partly responsive to prevailing interventions. ASC manifests core challenges in social skills, communication, and sensory function and by repetitive stereotyped behaviors, along with imbalances in the brain’s excitatory (E) and inhibitory (I) signaling. Repetitive transcranial magnetic stimulation (rTMS) has shown promise in ASC and may be a useful addition to applied behavioral analysis (ABA), a gold-standard psychotherapeutic intervention. We report an open-label clinical pilot (initial) study in which ABA-treated ASC persons (n = 123) received our personalized rTMS protocol (PrTMS). PrTMS uses low TMS pulse intensities and continuously updates multiple cortical stimulation locales and stimulation frequencies based on the spectral EEG and psychometrics. No adverse effects developed, and 44% of subjects had ASC scale scores reduced to below diagnostic cutoffs. Importantly, in PrTMS responders, the spectral EEG regression flattened, implying a more balanced E/I ratio. Moreover, with older participants, alpha peak frequency increased, a positive correlate of non-verbal cognition. PrTMS may be an effective ASC intervention, offering improved cognitive function and overall symptomatology. This warrants further research into PrTMS mechanisms and specific types of subjects who may benefit, along with validation of the present results and exploration of broader clinical applicability.

## 1. Introduction

Autism spectrum condition (ASC) is diagnosed in 1 in 36 children (US Centers for Disease Control), and it includes a broad array of core manifestations that include challenges in social skills, communication, cognition, and sensory function, along with rigid and repetitive stereotyped behaviors [1,2,3] and irritability and aggression [4,5]. The severity of each ASC core issue varies between subjects along a spectrum, and the pathogenesis of this syndrome is complex, involving about 600 confirmed genes and a plethora of epigenetic and environmental factors in conjunction with the involvement of stress, immune, glucoregulatory, and reward systems [6,7,8,9,10,11,12,13]. Consequently, the debate about ASC etiology in terms of a common final pathway versus various mechanistic subgroups is still ongoing [14,15,16,17].

Notwithstanding, a widely held model describing ASC’s pathophysiology involves the brain’s excitatory–inhibitory disbalance (E/I disbalance) [6,14,18] driven by impairments in the respective glutamate and GABA pathways and their interface with dopamine [14,19]. This disbalance is clinically manifested via frequently comorbid seizures that are attributable to hypersynchronous neuronal activity [20,21]. ASC dopaminergic abnormalities [15,16] are evident in ASC patients’ insensitivity to social [14] and nonsocial types of reward, that is to say, reward deficiency [11,22].

Currently, ASC pharmacotherapies modulate only ASC comorbidities, not core manifestations [23]. Applied behavioral analysis (ABA) is an intensive, long-term behavioral intervention that is quite effective and is widely regarded as the gold-standard ASC therapy [24,25,26,27]. ABA is based on direct and firm positive and negative feedback, although it has been criticized by some clinicians as being potentially demanding [28]. Moreover, ABA is time consuming and rather costly [26]. A rational goal, therefore, is to reduce the intensity and burden of ABA while maintaining its efficacy by adding a well-tolerated and cost-effective mechanistically orthogonal treatment modality that may potentiate ASC treatment outcomes.

A candidate therapeutic modality that is FDA-approved for major depression, obsessive-compulsive disorder, migraines, and smoking cessation, and is proving to have heuristic value for a range of neuropsychiatric disorders, including ASC, is repetitive transcranial magnetic stimulation (rTMS) [29,30]. Standard rTMS protocols commonly consist of daily rTMS therapy to a single cortical location, the dorsolateral prefrontal cortex (DLPFC). Stimulation intensity ranges from 80 to 120% of the motor sensory threshold or the threshold required to make a visible finger twitch and is delivered daily, 5 days a week, for approximately 30 min per day (Table 1). The current standard of care commonly delivers a frequency of 10.0 Hz to all patients, and this frequency does not change throughout the course of treatment.

Our team expanded the conventional technique to what we term personalized rTMS (PrTMS), which is based on the stimulation of a relatively larger portion of the brain cortical mantle, a general concept that has been suggested by numerous rTMS studies and position papers [31,32,33]. Contrary to standard rTMS protocols, PrTMS is not confined to one or two cortical sites but rather stimulates the DLPFC, orbitofrontal cortex, medial posterior cortex, and the central motor strip, thereby covering substantially larger areas and different functional territories of the cortex, against the backdrop of reduced TMS amplitude (power) [34].

Consequently, PrTMS engages brain regions that are therapeutically meaningful in ASC and that encompass key pathways associated with the default mode network (DMN), one of the most dysregulated brain networks in ASC [35,36,37,38]. The DMN processes information about the self, others, and the surrounding context [37,38,39]. Moreover, PrTMS engages the subthalamic nuclei and the thalamus, which communicate with the motor cortex and can thus modulate the so-called hyperdirect signaling pathway. This pathway circumvents the striatum and involves linkages between the motor cortex and the globus pallidus external, the subthalamic nucleus, globus pallidus internis, thalamus, and cerebral cortex, and reportedly underlies ASC-related deficits in social interaction, communication, cognition, and emotion [40,41,42].

PrTMS stimulation locales, frequencies, intensity, train length, and intervals, are identified via the spectral electroencephalogram (EEG), which guides the attempted restoration of alpha oscillatory synchrony across significant areas of the cortex. The goal is to re-establish normal alpha oscillations, regional activity, and inter-regional signaling in the cortical mantle, as manifested by the subject’s intrinsic resting alpha rhythms, and in accordance with an appropriate and healthy posterior to anterior alpha frequency gradient [43]. The use of lower pulse amplitudes in PrTMS avoids triggering seizures via overstimulation of the motor cortex [44,45].

The latter is also important because high intensity rTMS stimulation of the premotor and motor cortex in humans and animals seems to lead to inferior results, possibly due to long-term potentiation (LTP) threshold effects [46]. On the other hand, low intensity PrTMS improves outcomes owing to the induction of beneficial forms of neuroplasticity [46,47,48,49,50]. Hence, PrTMS is specifically designed to accelerate the therapeutic response to TMS stimulation, whereby treatment dose is gradually intensified and adjusted based upon serial review and analysis of changes to the spectral EEG pattern and changes in weekly psychometric scores. Stimulation frequency is selected via a proprietary algorithm (Peaklogic, Inc., San Diego, CA, USA) and adjusted according to cortical location such that 3–5 different frequencies may be employed in a single treatment session [44,45].

We have successfully applied the PrTMS methodology to patients with brain concussion and to those with post-traumatic stress disorder and neuropsychiatric syndromes that have several key manifestations in common with ASC, such as reward deficiency and E/I disbalance [44,45,51,52,53,54]. The purpose of the present pilot study was to administer PrTMS to ASC persons already receiving standard ASC therapy including ABA and elicit a positive therapeutic effect. We hypothesized that PrTMS would significantly reduce ASC manifestations in a substantial fraction of subjects. The aim was to acquire results that may justify follow-up with a more formal, prospective sham-controlled study. The utility of distinct spectral EEG changes as an objective marker for the clinical characterization of ASC was examined in an exploratory fashion.

## 2. Methods

### 2.1. Subjects

Subjects with a Diagnostic and Statistical Manual of Mental Disorders, 5th Edition, Text Revision (DMS-5-TR) diagnosis of ASC were recruited from the array of ten clinics participating in PrTMS protocols. After the procedures were fully explained, all subjects gave written informed consent to the protocol approved by the WCG Institutional Review Board (study number: 1254094; tracking number: 20190239). Despite the fact that these persons already had formal ASC diagnoses, routine psychodiagnostic/-metric assessments were conducted before and after PrTMS in order to provide a measure of PrTMS efficacy. Psychometric scales differed between clinics but included only two, **(1)** the Autism Spectrum Quotient (ASQ) and **(2)** the childhood autism rating scale (CARS) [55,56,57]. Both tests were administered by parents or caregivers after instructional briefing by clinical staff. Clinical assessments, including eligibility for TMS, EEG analyses, and PrTMS treatment, were conducted by trained physicians and medical technicians. All races, sexes, ethnicities, and socioeconomic levels participated in the study, and the demographics of the patient populations assessed by the ASQ and the CARS tests are presented in Table 2 and Table 3, respectively. Subjects were required to have stable medication and ABA regimes for at least 8 weeks prior to commencing PrTMS treatment study enrollment and to have satisfied well-established rTMS safety and exclusion criteria [58,59,60]. Exclusion was also based on the presence of a major psychiatric illness such as bipolar disorder, schizophrenia spectrum disorder, major depression, and drug/alcohol use disorder. Following the screening and selection of patients, a PrTMS procedure briefing was given, and all patients or parents/legal guardians provided informed medical consent for treatment. Patients continued their ABA therapy and medication(s) during PrTMS treatment.

### 2.2. PrTMS Regimen

PrTMS was given daily, 5 days per week, typically for 6–11 weeks with a range of 1–71. Participants were checked each day for adverse events (AEs), which included headaches, scalp pain, cognitive deficits, and seizures. AEs also encompassed observed or self- or parentally reported problems, complaints, physical signs and symptoms, new-onset medical conditions, and previous medical conditions that worsened. Adverse event severity was defined according to the following descriptors: mild awareness of discomfort but easily tolerated, moderate discomfort enough to cause interference with usual activity, or severe incapacitating discomfort with the inability to perform work or typical activities.

On Monday of each treatment week, which lasted 5 days, participants or their parents completed psychometric questionnaires, and the choice of questionnaire varied between participating clinics (Table 2 and Table 3). The EEG was recorded from seated subjects who were awake with eyes closed, using a 19-lead high impedance dry electrode EEG headset (Cognionics Inc., San Diego, CA, USA). Pre- and post-EEG acquisition processing steps were minimized, and the data were then normalized and rendered into waveform distributions in a frequency versus amplitude power spectrum. Commonly referred to as a “spectral EEG map or EEG mapping”, repeated EEG studies were obtained every 5–7 treatments, and treatment protocol and frequency adjustments were created, resulting in a renewed treatment plan. It was the patient’s own responses to stimulation that drove the treatment algorithm and protocol selection.

When the EEG could not be cleanly acquired in younger subjects [61], we referred to our previous experience with children to apply age-appropriate frequencies generally consistent with the published literature [62]. In such cases, subjects aged 5–7 years were treated with 9.4 Hz, subjects aged 7–10 years received 9.6 Hz, and those older than 10 years were treated with 9.8 Hz. We treated the dorsolateral prefrontal cortex and, in addition, targeted the central motor strip, the posterior frontal cortex, the orbital frontal cortex, and the right prefrontal cortex. Cortical locations that exhibited an alpha center frequency deviating from the subject’s predicted intrinsic alpha center frequency for that location were stimulated at the projected intrinsic frequency. These person- and region-specific intrinsic alpha center frequencies were as an initial approximation derived from occipital electrode spectral EEG records, since alpha generators occur in the thalamus and the visual cortex, which is occipitally located [63,64].

Following stimulus frequency selection, treatment was delivered by a trained rTMS technician using a MagVenture MagPro R30 transcranial stimulator and B-65 head transducer (MagVenture, Farum, Denmark). Patients remained seated without sedation in a quiet room and were asked to keep their eyes closed. The selected magnetic field intensity was initially low and was gradually increased over the course of treatment. Stimulation intensity was 25–60% of the resting motor threshold in most patients, and the stimulus frequency range was 8–13 Hz, with magnetic pulses delivered in 10–15 s trains. Intertrain intervals began at 30 s and gradually decreased to 10 s. During each treatment session, which lasted about 40 min, the motor-sensory strip and subsequent prefrontal and frontal regions were treated in succession.

### 2.3. Data Analysis

EEG data pre-processing included visual inspection and removal of distinctly erratic and technically flawed recordings identified by experienced technicians who were ‘blind’ to the study design and hypotheses. In line with the established procedures, filtering and selective removal of EEG recordings were avoided or minimized as much as possible in accordance with the views of de Cheveigné et al. (2019) [65]. A four-minute EEG time epoch was transformed via Welch’s fast Fourier transform (FFT) employing a custom Python program to produce a power spectrum with 0.1 Hz resolution; the spectral frequency band was restricted to between 2 and 20 Hz in the power spectrum. The extracted alpha band (8–13 Hz) power spectrum used in the subsequent analysis is devoid of low-frequency artifacts, obviating the necessity of filtering with consequent potential for bias. A proprietary spectral EEG analysis algorithm (PeakLogic, Inc., San Diego, CA, USA) identified an initial stimulation frequency in the alpha band and continually adjusted this frequency as a function of the change in objective alpha wave characteristics according to successive EEG power spectral acquisitions, and clinical response, as measured by the psychometric questionnaires.

The primary PrTMS efficacy endpoint was the reduction in symptoms measured by the ASC, CARS, or ASQ, acquired weekly from baseline (pretreatment) to the final treatment week. Treatment efficacy was defined as a statistically significant reduction in mean scores compared to baseline. Observed (raw) scores were summarized in terms of the number of non-missing observations (n), mean, standard deviation (SD), median, and range by time point. For the EEG analysis, the dominant alpha peak (center) frequency was determined for all EEG leads, averaged for each cortical electrode over all subjects, and a repeated-measures ANOVA compared data before and after PrTMs. The amplitude of the alpha band (8–13 Hz) spectral center frequency was identified for each EEG lead up to 10 weeks of treatment. For robust regression analysis, for each patient, the mean peak amplitude for the entire brain cortex was averaged for all responders and nonresponders for pre-PrTMS and weeks 6 and 10 of PrTMS. The 1/f^α^ aperiodic spectral component was plotted using the base 10 log of the 2–20 Hz frequency scale versus the log of the mean peak amplitude and then calculating the robust regression line, which treated periodic oscillatory components as outliers [66,67,68,69,70].

Community involvement statement. There was no community involvement in the reported study, although the senior author, Dr. Kevin Murphy, treated his autistic son with PrTMS for several years with remarkable success.

## 3. Results

### 3.1. ASC Psychometric Tests

**Figure 1:**
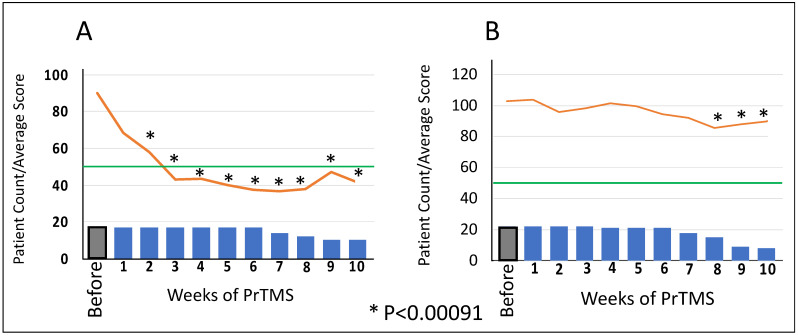
**PrTMS responders and nonresponders evaluated with the ASQ test.** Graph (**A**) on the left shows mean ASQ scores for PrTMS treatment responders. These patients were defined by ASQ scores that at some point over the multiweek PrTMS treatment course declined to or below the autism diagnostic cutoff score of 50. Graph (**B**) shows data for nonresponders, who were classified as patients whose ASQ scores did not decline to or below the diagnostic cutoff of 50 during the course of PrTMS treatment. Weeks of treatment are shown on the *x*-axis, and the mean test score and number of subjects tested each week are indicated by the vertical axis. The bars denote the number of patients, and the orange line is the mean ASQ test score. The green horizontal line shows the ASC cutoff score, where above 50 indicates ASC. For both graphs, A and B, the asterisks indicate significant differences via multiple comparisons and imposing the stringent Bonferroni correction for significance, α corrected = α/m = 0.05/55 = 0.0009091.

**Figure 2:**
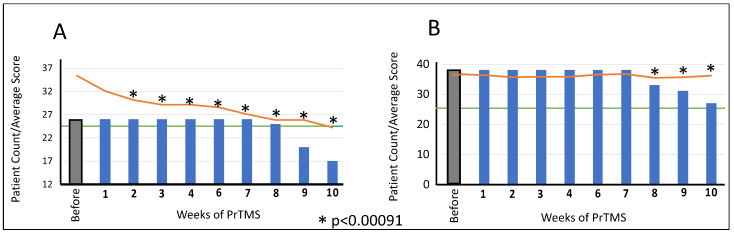
**PrTMS responders and nonresponders evaluated with the CARS test.** Weeks of treatment shown on *x*-axis and mean test score and number of subjects tested at each time point are indicated by the vertical axis. PrTMS treatment responders were defined as patients whose CARS scores at some point over the multiweek PrTMS treatment course declined to or below the autism diagnostic cutoff score of 25 as shown by Graph (**A**). Graph (**B**) shows data for nonresponders who were classified as patients whose CARS scores did not decline to or below the diagnostic cutoff of 25 during the course of PrTMS treatment. Graph (**B**) shows a mild CARS score decline in nonresponders during PrTMS. For both graphs, (**A**,**B**), the asterisks indicate significant differences via multiple comparisons with the Bonferroni correction for significance, α corrected = α/m = 0.05/55 = 0.0009091.

When evaluating the depicted results, here, it is useful to bear in mind that subjects were either evaluated via the ASQ or the CARS test, depending on at which of the 10 participating clinics they were being treated. Table 2 and Table 3 indicate how many participants were initially rated with each scale. The PrTMS response for individuals who were assessed with the ASQ psychometric test is shown in Table 2 and Table 3 and by Figure 1A,B. Results for those persons assessed by the CARS test are shown in Figure 2A,B. Both sets of figures generally show that by around week 7 of treatment, subjects dropped out of therapy, and it is important to note that as far as we were aware, these dropouts were not because of adverse treatment effects. The figures indicate for each week the subject count, which also indicates how many subjects were analyzed for that week. The data are partitioned into two groups for each of the ASQ and CARS cohorts, namely, responders and nonresponders. Patients were categorized as responders or nonresponders depending on whether their psychometric scores declined to or below the threshold cutoff for a diagnosis of autism at any point during their multiweek treatment course. The EEG was not used to classify patients as responders or nonresponders. Fluctuation was not taken into account, although over this treatment time period, most patients were stable. Responders in the patient group assessed via the ASQ test were defined as patients whose ASQ scores over the course of PrTMS treatment declined to or below the diagnostic ASC cutoff score of 50. Nonresponders in the ASQ-assessed group were classified as patients whose ASQ score did not decline to or below the ASQ diagnostic cutoff of 50 for autism during the course of PrTMS treatment. For the CARS-assessed patients, PrTMS treatment responders were defined as patients whose ASQ score at some point over the multiweek PrTMS treatment course declined to or below the autism diagnostic cutoff score of 25. These patients were no longer regarded as exhibiting autism according to the CARS test. Nonresponders were classified as patients whose CARS scores did not decline to or below the diagnostic cutoff of 25 during the course of PrTMS treatment. The ASQ responders had a median age of 19, while nonresponders had a median age of 13 (Table 2). CARS responders had a median age of 11 while nonresponders had a median age of 12 (Table 3). Figure 1A reveals that by 3 weeks of therapy, ASQ responders robustly showed an average score decline to below the autistic threshold of 50. This deep, early response was faster and greater than that seen with the CARS responders, who were younger.

Figure 1A indicates that over the first 10 weeks of treatment, the decline in average ASC score for responders was rapid, substantial, and statistically significant for all weeks, while Figure 1B shows a gradual decline in nonresponders that never reached the threshold of 50 but was nonetheless a partial response. For the CARS subjects, Figure 2A indicates that by week 10, the average CARS score fell quite rapidly below the threshold of 25, which was statistically significant, and by week 8 (7 weeks of PrTMS), subjects began leaving the study. Both Figure 1B and Figure 2B, which show non-responder data, indicate that over several weeks of therapy, some patients did not attain the autistic threshold. Although there was a decline in average scores, it was slow and shallow, suggestive of a partial response to PrTMS. Statistical significance was attained at weeks 8, 9, and 10 of PrTMS therapy.

**Figure 3:**
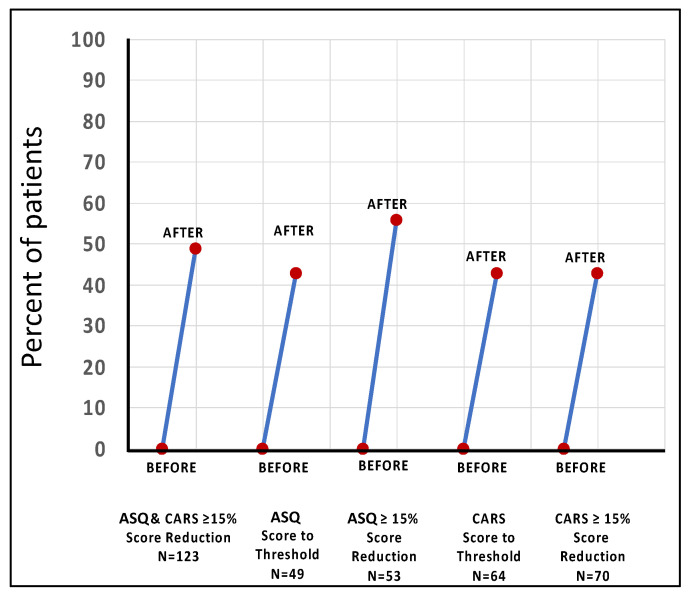
**Percentage of tested patients showing ASC psychometric score reductions to threshold or 15% or greater of pretreatment levels.** Data shown are for both ASQ and CARS tests. The total number of patients tested in each category is shown, and the percentage of that number responding before and after PrTMS is plotted on the graph.

The number of subjects that attained either autistic thresholds or exhibited a score decline equaling or exceeding 15% of the pretreatment score is shown in Figure 3. The proportion of all patients experiencing a 15% or greater decline in score was 49%, while for ASQ subjects, which were the oldest group with a median age of 19, this reached 55%, and for CARS subjects, who were younger, with a median age of 11, it was 44%. The population percentage attaining psychometric diagnostic threshold levels for ASC was 44% for both ASQ- and CARS-evaluated patients.

### 3.2. Spectral EEG

**Figure 4:**
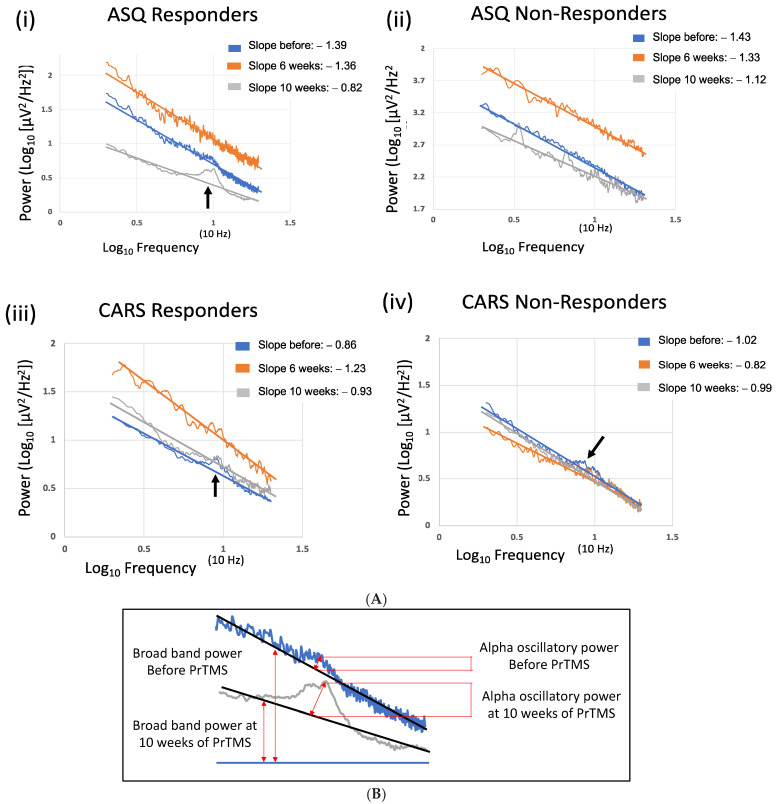
(**A**). **Robust regression analysis of mean spectral EEG.** Logarithmic spectral EEG plots and their robust regression lines denoting average aperiodic signals are indicated for each ASQ and CARS group. The logarithmic *x*-axis represents 2–20 Hz, and the *x*-axis number 1 corresponds to 10 Hz. Average EEG amplitude is indicated by the *y*-axis in log_10_(μV^2^/Hz^2^), and for each set of plots, the *y*-axis scaling is specifically adjusted to aid visualization. Robust regression slope numbers are tabulated in the upper right of each set of plots. The oldest age group shown in panel (**i**), the ASQ responders, had a notably shallower slope after 10 weeks of PrTMS, with the emergence of a clearly defined alpha peak (black arrow) with increased oscillatory alpha power, slightly beyond 10 Hz. EEG acquisition was challenging for this group and very difficult for all of the other groups. Panel (**ii**) ASQ nonresponders had greater aperiodic (broadband) EEG power than did responders, with a somewhat shallower slope at 10 weeks compared to pretreatment, and no clear alpha peak. Panel (**iii**) In CARS responders, the slope changed little, but there was a 10-week alpha peak below 10 Hz (black arrow) that was smaller in amplitude than pretreatment. Panel (**iv**) CARS nonresponders had little slope change, and the pretreatment graph shows an irregular alpha peak (black arrow), which apparently regressed after PrTMS. (**B**). **Alpha oscillatory power at 10 weeks of PrTMS in ASQ responders.** This enlarged section of the averaged spectral EEG for ASQ responders shows increased alpha oscillatory power above the regression line at 10 weeks. The 6-week recording for this group did not exhibit a discernable alpha peak, possibly due to difficulties associated with EEG acquisition in ASC patients, although, interestingly, broadband power for all of the groups tended to be comparatively high at 6 weeks as seen in (**A**), panels (**i**–**iii**), while for panel (**iv**), the before and 6-week recordings were close. This effect may have been in some way associated with the absence of a clearly defined alpha peak, although there were hints of such peaks.

The EEG was challenging to acquire with young ASC patients, i.e., median age of 11 to 13 years, as they tended to fidget and frequently opened their eyes, which extinguished or reduced the alpha peak. EEG recordings with the older subjects, ASQ group, median age 19, were more successful, and Figure 4A, which depicts the data for the older subjects, shows that before PrTMS, the average EEG was noisy and a distinct alpha peak was not clearly defined, although it was present. After 10 weeks of PrTMS, the average recording was smooth, and a very distinct alpha peak was discernable, with the highest point, i.e., center frequency, at 10 Hz. The size of the alpha peak substantially exceeded in scale the pretreatment alpha peak, denoting increased alpha oscillatory power.

The robust regression line for the spectral EEG log–log plot for older ASQ responders (median age = 19) was comparatively steep before PrTMS and became shallower by 10 weeks, suggesting less synchronicity and perhaps less inhibition and a greater diversity of signals. Moreover, the broadband power was reduced, although oscillatory alpha power was increased, and the graph was much smoother at 10 weeks (*p* < 0.05). Interestingly, young (median age = 11) nonresponding subjects whose EEG average spectra are depicted in Figure 4B, exhibited greater broadband power than responders at all timepoints, never attained a well-defined alpha peak, and their mean regression slope was less steep at 10 weeks. Increased broadband spectral power may suggest that ASC and inhibition were more pronounced in these subjects and/or that their ASC may have in some way differed in terms of its pathogenesis.

The CARS scale responder group, which was also young (median age = 11), did show an alpha peak after PrTMS, although the peak frequency was clearly below 10 Hz (Figure 4A(iii)). The robust regression line for the spectral EEG log –log plot by week 10 was slightly steeper, and the broadband power was about the same as the pretreatment level. These results may suggest that there may have been an age effect in CARS responders as they were significantly younger than the ASQ responders. The CARS nonresponders had week 10 spectral amplitudes that were lower than responders, and the pretreatment regression slope was steeper for nonresponders than for responders (Figure 4A(iii,iv)). The CARS week 10 regression slopes were very close between responders and nonresponders.

**Figure 5:**
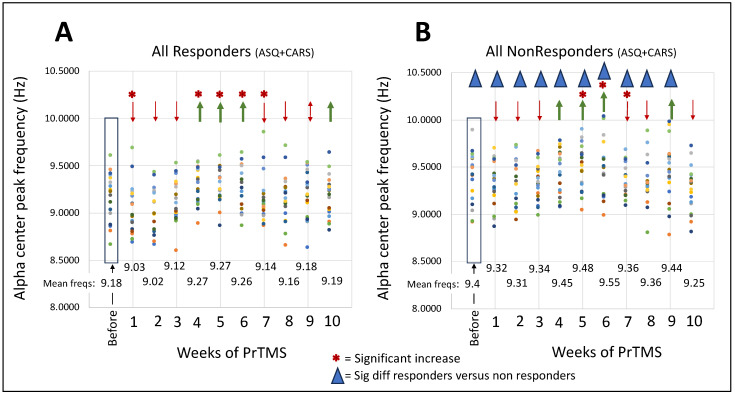
**Alpha peak center frequency before and after PrTMS.** The alpha peak center frequency was averaged for individual electrode positions amongst all subjects according to each week of PrTMS. Graph (**A**) shows the results for all responders, ASQ and CARS, and graph (**B**) shows the data for all nonresponders, ASQ and CARS. The mean alpha peak center frequency (MACF) is shown for each week of PrTMS. A repeated-measures ANOVA followed by pairwise *t*-tests indicated that the weekly means were not all equal. PrTMS treatment weeks showing significant decreases and increases in MACF are denoted by red asterisks. With both responders and nonresponders, the MCAF initially declined; then at 4–6 weeks, responders had increased MCAF; and interestingly, at 5 and 6 weeks, nonresponders, which as a group did exhibit a partial ASC psychometric response, also had increased MACF. Then, after the initial reaction, decrease, and increase in MCAF, with both groups, the MCAF subsided somewhat starting at around 7 weeks. Nonresponders versus responders had higher MCAF before PrTMS and at weeks 1–9 of treatment, indicated by blue triangles on graph B, derived from paired *t*-tests with the comparatively stringent Bonferroni correction.

The alpha band peak center frequency was averaged for each electrode for all of the responders grouped together (ASQ + CARS) and for all of the nonresponders grouped together (ASQ + CARS). These data are represented in Figure 5. Both responders and nonresponders exhibited statistically significant alpha center frequency increases between about 4 and 6 weeks, although this increase did not persist to 10 weeks. Some nonresponders exhibited a slow, shallow reduction in ASQ and CARS scores during PrTMS, as seen in Figure 1B and Figure 2B, so the emergence of increased alpha frequency with PrTMS in what we call nonresponders is not entirely unexpected. It may be significant that nonresponders generally exhibited higher alpha center frequency compared to responders, before and after PrTMS, as validated by paired *t*-tests between the two patient groups for pretreatment, and for weeks 1–9 of PrTMS. We used the Bonferroni correction as a stringent constraint on the *p*-values. For the younger age groups, with whom EEG acquisition was difficult, these results may contain artifacts because many patients did not have a discernable alpha peak; hence, identification of a peak alpha frequency via the automated software system we used may have been subject to inaccuracies.

**Figure 6:**
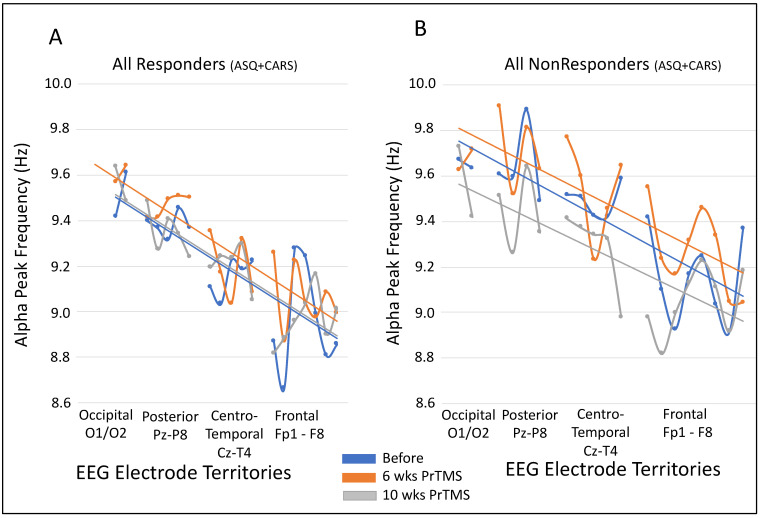
**Mean alpha peak center frequency according to cortical region.** The alpha peak center frequency was averaged for all subjects and for all electrodes in each of four EEG cortical brain territories before PrTMS and at 6 and 10 weeks of treatment. Blue lines indicate before PrTMS, while orange and gray indicate 6 weeks and 10 weeks, respectively. Trend lines are shown in respective colors. Graph (**A**) shows the results for all responders, ASC and CARS, and graph B shows the data for all nonresponders, ASC and CARS. In graph (**A**), the occipital mean frequency for responders remained close before and after PrTMS, indicating the stability of this region as an alpha generator relative to the frontal cortex. There is a progressive steep downward trend anteriorly. In graph (**B**), it is clear that for nonresponders, there is also a downward frequency trend, but it is shallower, and the data and trendlines are more widely dispersed.

The alpha center frequency averaged for each of the four main brain areas from poster to anterior exhibited a clear and expected posterior to anterior progressive gradient in alpha center frequency, as depicted in Figure 6A, with the highest frequencies occurring at the occipital electrodes, i.e., the visual cortex. The alpha frequency averages for responders and nonresponders both include ASQ- and CARS-identified patients. A decline in the alpha center frequency gradient progressing anteriorly was also recorded for nonresponders, as shown in Figure 6B, but the data are more dispersed, and the timepoint trendlines are more widely separated. This is a clear difference between responders and nonresponders, and the trendlines of the alpha center frequency gradient are steeper and much closer in responders compared with nonresponders. In both Figure 6A,B, the occipital center frequencies between before and after PrTMS are relatively close compared with other cortical regions, highlighting the stability of the visual cortex as a key generator of the alpha rhythm in the brain, and the less organized appearance of the graphs for responders suggests less coordination between cortical oscillators and perhaps less inhibition of some types of neural activity.

## 4. Discussion

This report describes the results obtained with PrTMS treatment of ASC patients in our clinic. The superiority and safety of the lower-power TMS approach exposing more of the cortical mantle to the stimulating magnetic field are suggested by a growing body of evidence [31,32,33,34]. None of the subjects developed seizures or reported any other side effects, and PrTMS induced the most rapid and marked responses in the oldest age group, viz., ASQ responders (median age = 19), with 55% of subject scores showing a reduction of 15% or more, and 44%, called ‘responders’, exhibiting a reduction to or below the ASC diagnostic threshold. This may have been a result of the greater relative ability of older patients to sit still under the rTMS head transducer, or it may reflect differences in brain development and/or ASC status. Younger patients (median age = 11–13) did in fact respond well to PrTMS as they also received stimulation to multiple cortical sites; 44% of CARS-identified (young) ASC subjects experienced score reductions of 15% or more, and 44% were responders, with score declines to or below the diagnostic threshold. Even though it may be possible that older subjects possessed a wider appreciation of the entire treatment process and thus may have been more susceptible to placebo effects, seemingly nonsubjective changes in the spectral EEG imply that the greater improvement seen with older subjects may not have been entirely derived from placebo.

The spectral EEG alpha center frequency initially decreased and then increased above pretreatment for the ASQ and CARS responders (median = 19 yrs), while the ASQ and CARS nonresponders, who as a group did display a partial response to PrTMS, also exhibited these alpha frequency changes. The alpha center frequency has been found to exhibit a strong positive correlation with non-verbal cognition scores in ASC children [71,72]. All the responders and nonresponders (ASQ+CARS assessed groups) had alpha center frequencies that initially dipped and subsequently rose to peak between weeks 4 and 6 of PrTMS, and then they slowly declined. This may have been coincident with some comparatively transient form of activation or reorganization of cortical signaling contributing to the alpha peak and the center frequency, although the true mechanism and its possible relationship to the substantial clinical improvement seen in responders is not clear. Nonresponders did exhibit a partial response in terms of psychometric scores, and how this correlated to the observed variations of the alpha center frequency may be the subject of future investigations. The spectral EEG broadband power was higher in ASQ nonresponders than responders. Moreover, collectively, ASQ and CARS nonresponders had substantially higher mean alpha peak frequency both before and after PrTMS treatment compared to ASQ and CARS responders as a single group, and this difference was statistically significant. It is possible that nonresponders had a higher degree of cortical excitation than did responders.

The spectral EEG logarithmic robust regression became flatter after 10 weeks of PrTMS in ASQ responders and slightly flatter after 10 weeks of PrTMS in ASQ nonresponders. This effect was not observed for CARS responders and nonresponders. This potential marker deserves follow-up to determine its validity as it may be important in the context of the role of E/I balance in ASC pathogenesis and, here, may have been associated with reduced inhibition of neural signaling in the brain cortex, as suggested by various authors [66,67,68,69,70]. On the other hand, the presence of an alpha peak may be associated with greater inhibition of some types of cortical activity [73]. The older ASQ responder group displayed the emergence of a substantial alpha peak above the broadband level of the spectral EEG, i.e., increased alpha oscillatory power was recorded (see Figure 4A). This suggests that more neurons in the cortical mantle were recruited to oscillate at frequencies within the 8–13 alpha band range, possibly indicative of greater cortical oscillatory synchrony [74,75]. However, it may be important to note that nonresponders were younger, and alpha power is higher in younger versus older subjects [76].

Younger subjects, median age = 11–13 years, did not exhibit the evolution of a well-defined alpha peak or smoothing of the spectral graph, likely because these patients tended to open their eyes during EEG acquisition and often moved about the treatment room, thereby markedly reducing the alpha peak [77]. Young subjects also fidgeted under the treatment head transducer which may have affected treatment efficacy; though the young CARS responders exhibited significant reductions in CARS scores. We cannot rule out the possibility that the appearance of an alpha peak in older subjects versus younger individuals was not artifactual and may have indeed reflected neurobiologically based differences between age groups.

A limitation of this study is its open-label design, which carries a risk of bias, particularly among the older ASQ responder age cohort. This group was likely more cognizant of the treatment and its potential effects. However, despite such potential bias, notable changes were observed in the spectral EEG parameters, e.g., flattening of the EEG spectral regression and an increase in the mean alpha center peak frequency. Although it is conceivable that some subjects may have experienced psychological benefits from PrTMS and the accompanying medical attention reflected by the EEG effects, such EEG changes would not typically arise from placebo effects, especially over 10 weeks. Another limitation lies in the difficult nature of EEG acquisition in younger age groups, who also proved challenging to treat with TMS. Despite these obstacles, the overall spectral EEG results suggest that the spectral EEG warrants further investigation as a hitherto unavailable [78,79] objective neurophysiological ASC biomarker not only applicable to PrTMS but also to ABA and other therapies. Obviously, more studies are warranted in terms of examining further the spectral EEG as an objective measure to enhance our understanding and management of ASC. Finally, despite the fact that no adverse events were observed, it is possible that targeting multiple cortical sites in order to stimulate wider areas of the cortex may present disadvantages. For example, normal brain networks may be interfered with, and stimulation of broader areas may create overlapping effects that could confound interpretation. Moreover, it is entirely possible that as more is learned about the discrete brain regions that potentially drive autism, it may be determined that stimulation of extraneous sites may (1) act to diminish the therapeutic effects of specific targeting and (2) complicate the identification of discrete nodes and pathways specific to each autistic subject.

In summary, in this pilot study, individualized PrTMS treatment of ASC patients led to significant improvements in psychometric scores and discernable changes in spectral EEG parameters without adverse events. The spectral EEG may emerge as an objective biomarker of ASC. To address the complex needs of ASC patients, it is important to continue this line of inquiry through formal studies aimed at validating our results to enhance the rather limited therapeutic armamentarium currently available for treating ASC and to gain further understanding of the neurophysiological underpinnings of ASC.

## Figures and Tables

**Table 1:** Differences between standard rTMS and PrTMS.

Parameter	Standard rTMS	Personalized rTMS
EEG	None	Every 5–7 treatments
Spectral EEG analysis	None	Every 5–7 treatments
Cortical region treated	DLPFC	Multiple locations
Stimulation frequency	10 Hz	Personalized, 3–5 diff freqs/patient
Stimulation level (% motor threshold)	120%	Reduced intensity/dynamic/custom
Trains/session	30	Dynamic/custom
Train length	6 s	Dynamic/custom
Interval length	54 s	Dynamic/custom

**Table 2:** ASQ demographic data.

All	Before PrTMS	Treatment Week 4	Before PrTMS	Treatment Week 6
Total subjects	53	53	43	43
Average score	97	75	96	73
Number of males	40	40	33	33
Number of females	13	13	10	10
Age range	1–34	1–34	1–34	1–34
Average age	16	16	16	16
Median age	17	17	18	18
Responders only	Before PrTMS	Treatment Week 4	Before PrTMS	Treatment Week 6
Total subjects	17	17	17	17
Average score	90	43	90	43
Number of males	14	14	14	14
Number of females	3	3	3	3
Average range	1–30	1–30	1–30	1–30
Average age	18	18	18	18
Median age	19	19	19	19
Nonresponders only	Before PrTMS	Treatment Week 4	Before PrTMS	Treatment Week 6
Total subjects	22	22	21	21
Average score	103	98	105	100
Number of males	16	16	15	15
Number of females	6	6	6	6
Average range	4–34	4–34	4–34	4–34
Average age	14	14	14	14
Median age	13	13	10	10

**Table 3:** CARS demographic data.

All	Before PRTMS	Treatment Week 4	Before PrTMS	Treatment Week 6
Total subjects	70	70	70	70
Average score	36	33	36	33
Number of males	55	55	55	55
Number of females	15	15	15	15
Age range	0–61	0–61	0–61	0–61
Average age	13	13	13	13
Median age	11	11	11	11
Responders only	Before PrTMS	Treatment Week 4	Before PrTMS	Treatment Week 6
Total subjects	26	26	26	26
Average score	35	29	35	29
Number of males	16	16	16	16
Number of females	10	10	10	10
Average range	4–44	4–44	4–44	4–44
Average age	12	12	12	12
Median age	11	11	11	11
Nonresponders only	Before PrTMS	Treatment Week 4	Before PrTMS	Treatment Week 6
Total subjects	38	38	38	38
Average score	37	36	37	36
Number of males	33	33	33	33
Number of females	5	5	5	5
Average range	0–61	0–61	0–61	0–61
Average age	13	13	13	13
Median age	12	12	12	12

## Data Availability

All data in this study may be obtained by contacting the corresponding authors.
